# Address of J. McF. Gaston, M. D., before the Graduates of the Southern Medical College, April 3d, 1895

**Published:** 1895-04

**Authors:** 


### ADDRESS OF J. McF. GASTON, M. D., BEFORE THE GRADUATES OF THE SOUTHERN MEDICAL COLLEGE, APRIL 3, 1895.

The invitation of our president to relieve him of a part of his onerous duties on this occasion was accepted in view of the fact that he had labored incessantly during the closing session of our college, not only filling his own hours, but taking the hours of every other member of the faculty who would consent to yield the time to him. I am under special obligation to him for taking my place, while I was off duty for a month, suffering from the despotic grasp of the grippe.

The great controlling influence with me was, that my venerable chief should be relieved of his burden by a younger member of the faculty. He and I were students together many years ago at the University of Pennsylvania; but it should be remembered that he was one of the oldest and I was one of the youngest in the class. Thus it turns out that with the motto, *virtus in arduis*, he is working still, under the accumulation of years, [redacted] and honors. In the meantime, I am renewing my youth with the multiplicity of various duties devolving on me; and we are both striving, in season and out of season, to keep up with this progressive age and our youthful colleagues.

A comparison of the facilities for medical education half a century ago, which passed under our observation, and were noted by us in due form, with what preceded that era, and what has followed, may prove profitable to the graduating class.

My limited time was to be occupied with a strictly professional view of this period, as my distinguished friend who was assigned the duty of making the general address on this occasion, was expected to afford a feast of reason in discussing matters of general interest to this intelligent assembly of ladies and gentlemen. A large proportion of those here to-night have undoubtedly been attracted by the reputation for erudition and eloquence of the Hon. W. C. Glenn, but indisposition prevents his appearance this evening. Not being capable of entertaining this audience with the rich cullings of literature, I shall content myself with briefly noting some matters for the guidance of those who are about to enter upon the practical work of treating the ills which flesh is heir to in this world of pain and suffering.

It may not be out of place at the outset to note that our small list of graduates on this occasion is due, as already stated by the Dean, to the inauguration of a three years' course of lectures, with six months for each session, for graduation in the Southern Medical College.

Formerly our students received the degree of M. D. after two sessions of five months each, and by great diligence and painstaking many of them have attained a fair measure of proficiency in their preparation for the practical work of the physician. But it is evident that by a graded course of three years, in which the first sesssion is devoted chiefly to rudimentary branches, that more satisfactory progress is made subsequently in the higher order of medical education.

The old fathers of medicine in by-gone days, such as Hippo crates, Boerhaave, Galen, Harvey, and their followers, were supposed to know all that was available of the curative art, and indeed, gave a stimulus to subsequent investigators of great importance. But their work reminds me of a Thompsonian practictioner who flourished in my section during my boyhood days. He had a mania for doctoring his patients with lobelia and Cayenne pepper, while they were immersed in hot water baths, in the presence of an old negro woman, who became quite an adept in all the details of the practice. On one occasion when Toney, the husband of the woman, was expatiating upon her great achievements, he remarked with emphasis: "Bless my soul! Mars Dan'l done teach Fanny more than he know hisself." So it was with those old worthies of early times; they taught the generation which succeeded them more than they knew themselves. At that period all was empirical in medicine, and little was attempted in practice which did not have the sanction of high authority. But gradually the professional dictum came to be set aside, and the physician began to look around him for new fields of investigation. With Harvey's discovery of the circulation of blood a new light dawned upon the medical world, and the fact was impressed upon the observers of results that they were not bound down by the records of the past. Independence of thought and action led to individual investigation; and "thus saith the record" was replaced by a large accumulation of observations in the different fields of medicine.

In the course of . events there came forth Bell, Sir Astly Cooper, Goode, Cullen, Marshal Hall, Velpeau, Physick, Valentine Mott, and others no less original and progressive in medicine and surgery.

Among the earliest organized movements for promoting medical education in this country stands the time-honored University of Pennsylvania. It was my privilege to receive instruction there from Chapman, Horner, Jackson, George B. Wood, Gibson, Hodge, and Hare, who were held in high esteem by the large class of students who attended their lectures at that early day.

This was the medical Mecca to which pilgrims flocked from all parts of the United States, and also from other portions of the world, being the great center of medical instruction.

Being impressed with the vast field for advancement in medical knowledge offered at Philadelphia, yet with that State pride which predominated in everything at that day, I was influenced to return to the Medical College of South Carolina to take my second course of lectures, and receive my degree of M. D. A faculty no less distinguished than that of the University of Pennsylvania filled the different professorships at that time in Charleston; and the faithful teaching of Dickson, Geddings, Holbrook, Moultrie, Frost, Prioleau and Shepard are vividly recalled by me. In purity and eloquence of diction, the lectures of Dr. Dickson on the practice of medicine have never been excelled; and he was afterwards called to fill the same chair in the Jefferson College of Philadelphia, sustaining fully his high reputation.

Notwithstanding the celebrity of these distinguished professors, which placed them far in advance of the false theories and vain speculations, of olden times, it is evident that they had utterly failed to grasp all the principles which lay at the foundation of true progress in teaching.

The advances which have been made in the past fifty years in all departments of medical science, are so stupendous, that should a professor of any branch taught in the curricula of that period be called forth to observe the course of instruction in the better class of our schools, scattered broadcast over this vast field of progressive education, he would stand aghast at the advancement. If the attainments for graduating with the title of M. D. formerly, were compared with the achievements of those receiving the degree of M. D. from our schools of high grade at the present day, the latter-day graduates would be proved to have far greater proficiency in all that pertains to the discharge of their professional duties. I don't hesitate to say that a sufficient number of graduates from the Southern Medical College could be selected each year to fill the professorships in every department more satisfactorily than they were filled by those holding these chairs fifty years ago. Yet it must be remembered that you are only upon the threshold of true progress. If any of us have gained recognition in the medical profession, it has been by dint of diligence in study after graduation, and I would urge upon each of you higher efforts after graduating.

It has been found most conducive to progress in practical attainment for the graduate to spend the first year or two after receiving his degree of M. D. as interne of a well regulated hospital, and these positions are considered so desirable, that some of our medical colleges make the assignment as internes to a hospital, prizes for those who have the best records upon examination. The importance attached to the advantages of securing these appointments is manifested in the number of graduates from our college who have been assigned to duty as internes in the Grady Hospital. My advice to the members of the present class is that any of you who can make it convenient to spend at least one year in this work should take the requisite examination for securing one of those places. I am pleased to note that the Grady Hospital is a well conducted institution.

That grand element of advancement in knowledge offered by the Polyclinic or Post-graduate schools, should enable every new-fledged doctor to apply the instruction which has been received in the medical college, and those who graduated under the old regime had not the facilities for the practical observation of the treatment of medical and surgical patients which are presented in the well organized and splendidly equipped hospitals of the present day. While much has been accomplished by faithful working in laboratories of different kinds and by experiments upon living animals, for the elucidation of biological problems, accurate observations of the progress of disease in the human subject and making records of their data for future reference is far more important to the practitioner.

It is desirable for the graduates to realize that, in receiving their diplomas, they have not learned all that is requisite to acquire for the practice of medicine. In other words, they do not know it all, and it is important that they should know their ignorance. It is thus essential to get a certain amount of knowledge to understand your shortcomings, and to appreciate what further must be learned to fit the graduate in medicine for the proper exercise of his professional duties. You are taught in college all the fundamental principles which are requisite for the degree of M. D., and to qualify the physician to enter upon his work in accordance with the laws of the land. He is fitted thus to take charge of the treatment of cases of diseases, and yet he must inform himself as to what has been done by others, under similar circumstances.

Some of you may have read the record of the experience of the lamented Dr. Marion Sims, upon first entering upon the practice of medicine, and his trials and tribulations in undertaking the treatment of his first patient. We must presume that he was well equipped with the teachings of the professor of practice in the medical college where he received his degree of M. D., and yet he was at sea completely in applying this instruction to the case in hand. After reading all the books which were available, his mind was in a state of confusion worse confounded, and he called in an experienced practitioner, to help him out of his dilemma. With similar results in other cases, he became so much disgusted that he resolved to leave that location, tore down his sign, and went to another section of the country. His efforts, after leaving South Carolina, and going to Alabama, were crowned with more success, and, by perseverance, with close application, he ultimately attained such distinction, as to be unequalled by any other member of the profession, at home or abroad.

This affords encouragement to all who may encounter difficulties at the outset, to redouble their energies in overcoming the obstacles which may be encountered, and to continue their studies by reading the best authorities in the different departments of medicine and surgery, after entering upon the pra c tical work of treating diseases. Not only should you study books, but observe closely the practice of experienced physicians, and elicit all the information possible, by getting their advice in consultation. It will not suffice to note the practice of others, but the graduate should study his own cases, and read every available book upon the disease under treatment, so as to select the very best remedies for the case in hand. All the accumulated experience of the best authorities in medicine and surgery is printed in a condensed form, for the information of the members of the profession, who are entering upon the untried field of practice, and you should not grope in the dark, when the light may be turned on, by simply opening the appropriate volume. Do not fail to exercise your rational faculties in determining what is best to do, after you have consulted the highest authorities in the matter under consideration, and each must be his own judge for the occasion.

In a consultation, a few years ago, with a prominent practitioner, who had been engaged several years in the duties of his profession, he remarked to me that he envied my experience as a physician and surgeon. In reply, I told him that all which had been acquired by observation, during a rather eventful life, would most cheerfully be exchanged for the chance to know, with his age, and the opportunity to profit, in future years, by what has been recently brought to light.

You have, then, not simply to pluck the fruit which is ripe, but to take advantage of the growing crop, to reap a magnificent harvest in the future. What those who have gone before you secured by hard work, by day and by night, is laid before you, to be turned to account in the developments of the near future.

The gradual but steady enlargement of the field of medical investigation, corresponds to the tension of a watch spring, which sets out with a small coil, and by subsequent turns, grows larger and larger, and extending from the outer circle, it is connected with the intricate machinery to keep it in motion.

The developments have gone forward, in the past two decades, with a rapidity which encourages the belief that still more valuable accessions will be made to our practical knowledge, within the next quarter of a century, and those members of the profession are to be congratulated who may have the satisfaction of observing the fully-completed work of this period. The triumphs of the surgery of the brain, the thorax, the abdomen, and the vascular system, will most probably be overshadowed by the magnificent advances in other branches of medicine and surgery.

The interminable list of remedies within easy grasp of the physician by the tact of manufacturers, continues to grow from year to year, and the diagnosis of diseases, through the implication of the nerve centers promises good fruits for the practice of medicine. But the present interest in bacteriology manifested by investigations of the laboratory and by clinical study offers the best prospect of working out good results of a practical nature at no distant day. The relation of cause and effect has, thus far, not reached a point which enables the pathologist to trace certain disorders to a bacterial origin, and the most that can be claimed is the connection of specific forms of disease with definite organisms or with microbes of a distinctive character. But there is hope for the future of bacteriology.

It is a gratifying reflection for the medical philosopher and scientist to point out the separate germs of the various disintegrating processes involved in the aggravated condition of wounds, and to define the special microbes of distinct diseases; but the practitioner is not always prepared to accompany the bacteriologist in such investigations, nor does it devolve upon him to accept the conclusions from his investigation for the application of remedies in surgical cases. After a somewhat careful research of what has been accomplished by the use of the microscope in detecting the different forms of micrococci and noting the discrepancy in the observations of different experts in this department, I am still in doubt as to the role of bacteria. But I am convinced that such micro-organisms and ptomaines have a share in the degeneration of vital structures.

My attitude in regard to the germ theory of diseases has not always been properly understood, but I wish it to be known that the results of investigations with the microscope are fully adpreciated, and that the observations of those who are experts in the use of this instrument are accredited as realities. There is no doubt in my mind of the existence of the comma bacillus in cholera, the bacillus tubercolosis, the cryptococcus xantogenicus in yellow fever, the anthrax bacillus in malignant pustule, and other specific germs which have been recognized as âccompanying various other forms of special diseases. But this is quite distinct from the proposition to combat these diseases by the use of germicides or to employ agents of this class as prophylactics against the development of disease.

I am on record as stating that all the practical appliances of surgery may be appropriately classed nnder the headings of Aseptic, Antiseptic and Septic processes. The first embody all those measures which come under the class of prophylactics, and look to the avoidance of hurtful agencies, thus being assured if the measure does no good, it will do no harm. As the term implies, asepsis consists in shunning all kinds of contamination from within and without the body. Antiseptic application in surgery differs widely from aseptic measures in their aim and results. The practice of antisepis is confined to correcting any tendency to or the development of degeneration and disintegration of living structures, and yet the claim for antiseptics includes measures which, instead of protecting the tissues, lead to septic development.

The employment of antiseptic vapors to correct the supposed vitiated condition of the air surrounding a patient, has long been exploded, having received its death blow from Thomas Keith, at the meeting of the International Medical Congress, in London. Antiseptic washes in operations upon normal tissues have been abandoned by many who formerly used them, while a large number of the profession, like myself, have never employed them, and are sustained by reports of the most advanced bacteriologists, that they are not simply innocent, but hurtful, by necrosing the healthy tissues. It is held that antiseptics should be reserved for those cases in which septic processes are to be combatted, and that antiseptic surgery ought to be limited to cases presenting septic conditions. Aseptic surgery has supplanted the so-called antiseptic surgery, in most surgical cases.

In accordance with the accredited doctrine of the Rev. John Wesley, that "cleanliness is next to Godliness," the medical profession has adopted the most stringent regulations against filth of every kind, and eschews dirt in all its forms, in surgical procedure, and in the regime of the sick room. Not only is the surgeon and physician, as well as the nurse to be scrupulously clean, but everything about the patient and bed must be kept clean.

The funeral procession of so-called antiseptic surgery is accompanied by a host of mourners, headed by Sir Joseph Lister, and the last rites of the solemn service at the tomb are to be conducted by a white-robed priest of the order of Esculapius, who with clean hands will lay the defunct body to rest, in everlasting oblivion. "*Requiescat in pace.*"

---

THE CURETTE IN PUERPERAL INFECTION.—Ferre (*Nouvelles Archives d'Obstetrique et de Gynecologie,* November 25, 1894) strongly advocates the use of the curette in puerperal infection, after a long experience of irrigation of the uterine cavity, a procedure which lowered mortality but did not save several bad cases. At the same time he did not have recourse to the curette after labor except when placental remains required removal. Since using the curette six bad cases had been treated by Ferre with only one death. The fatal case, it must be noted, was a private patient, and symptoms of infection immediately followed natural labor at term. She was left without assistance for five days, and the curette was employed as a last recourse. The patient died on the seventeenth day. In a second private case the curette was used on the second day immediately after a rise in temperature with rigors. The symptoms of infection at once vanished. In a third, a live child was born; a twin then presented by the shoulder. Embryotomy had to be performed. Fever set in on the same evening; next day large blount curettes were used, without anesthetic, the uterine cavity was swabbed with glycerin of creosote and plugged with iodoform gauze. All bad symptoms disappeared at once. The three remaining cases were in the Pau Lying-in Hospital, and had all the advantages of treatment in a public institution. They resembled the second above described, except that in one case parametritis set in before the curette could be used. All recovered.

